# Potent Antimicrobial Chloroindium(III) Phthalocyanine Sensitizer Targeting Drug-Resistant Microbes: Physicochemical, Photobiological Validation and DFT Insights

**DOI:** 10.3390/pharmaceutics18070874

**Published:** 2026-07-17

**Authors:** Aleksandra Pawska, Aleksey E. Kuznetsov, Marianna Szczepaniak, Daniel Ziental, Emre Güzel, Lukasz Sobotta

**Affiliations:** 1 Poznan University of Medical Sciences, Department of Inorganic and Analytical Chemistry, Rokietnicka 3, 60-806 Poznan, Poland; aleksandra.pawska@ump.edu.pl (A.P.); marianna.szczepaniak@student.ump.edu.pl (M.S.); dziental@ump.edu.pl (D.Z.); 2 Poznan University of Medical Sciences, Department of Genetics and Pharmaceutical Microbiology, Rokietnicka 3, 60-806 Poznan, Poland; 3 Department of Chemistry, Universidad Técnica Federico Santa María, Av. Santa María 6400, Vitacura, Santiago 7660251, Chile; 4 Department of Engineering Fundamental Sciences, Sakarya University of Applied Sciences, 54050 Sakarya, Türkiye; eguzel@subu.edu.tr

**Keywords:** indium(III) phthalocyanine, sensitizer, singlet oxygen, sonodynamic therapy, photodynamic therapy, density functional theory

## Abstract

**Background/Objectives:** An evaluation of the sensitizing properties of chloroindium(III) phthalocyanine complex (InPc) bearing 4-sulfonylphenoxy groups was performed. **Methods:** The ability to form singlet oxygen under light exposure was assessed, and the quantum yield Φ_Δ_ was calculated to be 0.82 ± 0.04. Under ultrasound exposure of the sensitizer (1 MHz, 3 W, 40% duty cycle), significant 1,3-diphenylisobenzofuran decomposition was observed. **Results:** Moreover, the macrocycle was assigned to be a moderate–high photo- and sonostable sensitizer. Density functional theory studies supported experimental results, suggesting the InPc to be a good photochemical agent. From the global reactivity parameters analysis, it can be suggested that InPc would interact easily with electron-excess species, such as various free radicals, in the solution phase, and should also be able to interact with electrophilic species. **Conclusions:** Studied InPc revealed high photodynamic antimicrobial activity and reached >4 log_10_ reduction in microbial growth against methicillin-resistant *Staphylococcus aureus*, and 4.08 ± 0.29 log_10_ against *Candida albicans* resistant to fluconazole (for dosimetry of 100 μM and 50 J/cm^2^). Interestingly, the photosensitizer studied was inactive against extended-spectrum *β*-lactamase-producing *Escherichia coli*.

## 1. Introduction

Photodynamic therapy (PDT) is a therapeutic approach that so far has shown significant efficacy against various disorders, mostly cancer and bacterial infections [1,2,3,4,5,6]. The core mechanism of PDT involves the administration of a photosensitizer (PS), which, upon activation by light of a specific wavelength, generates reactive oxygen species (ROS). These ROS induce severe oxidative stress, leading to bacterial death [3]. The potential of PDT depends largely on the photophysical properties of the PS, where an ideal PS exhibits, among other properties, high singlet oxygen quantum yields, strong absorption within the therapeutic window (650–800 nm), photostability, selectivity, and low dark toxicity [7,8].

Separately from PDT, sonodynamic therapy (SDT) has emerged as a therapeutic modality that utilizes ultrasounds (USs) to activate sonosensitizers (SSs) in target tissues, leading to ROS generation [9,10]. While SDT shares mechanistic similarities with PDT in terms of ROS-mediated cytotoxicity, it benefits from deeper penetration due to the nature of USs. Recent studies have suggested that certain PSs, such as phthalocyanines, can also act as effective SSs, expanding their potential applications beyond PDT [11,12,13,14,15,16].

Phthalocyanines (Pcs) are among the most promising classes of PSs due to their favorable structural and photophysical properties. They are widely recognized for their chemical and thermal stability [17,18]. The π-electron delocalization enables Pcs to exhibit strong absorption bands in the near-infrared region of the electronic spectrum, reaching high extinction coefficients (10^5^ M^−1^ · cm^−1^), along with high singlet oxygen quantum yields [19]. Generally, high triplet quantum yields with long lifetimes are obtained when Pc and its derivatives are complexed with closed-shell, diamagnetic cations like Zn^2+^, Al^3+^, Ga^3+^, In^3+^, and Si^4+^. Notably, indium phthalocyanines are paramount due to several key characteristics enhancing their efficacy and applications [2,11,12,13,20]. The significance of indium phthalocyanines in PDT stems from their advantageous photophysical properties, high potential to generate ROS, enhanced stability, limited tendency to aggregation, and potential for targeted delivery, all of which contribute to their effectiveness and appeal in antimicrobial photodynamic therapy (aPDT) [21,22]. Indium phthalocyanines demonstrate strong absorption in the therapeutic light window. This maximizes tissue penetration, enhancing the efficacy of PDT. Also, water-soluble Pcs have gained significant attention in PDT due to their unique properties [23]. The solubility of Pcs in aqueous environments ensures effective delivery to target tissues in PDT. It is particularly noteworthy that a combination of several sulfonated phthalocyanines has been utilized as Photosens^®^ for clinical PDT and in the diagnosis of cancer [24,25].

On the other hand, there are a few ongoing trials for the usefulness of sulfonated phthalocyanines for various infections [25,26,27,28]. Furthermore, anionic sulfonated Pcs are advantageous and serve two goals: they boost the effectiveness of treatment [29], and the negative charge causes the mutual repulsion of the phthalocyanine rings, which makes them soluble as monomers in water [30]. Considering these properties, the research has focused on water-soluble PSs as a strategy to employ Pcs in conjunction with the anionic sulfonated group to perform photophysical, photochemical, and sonochemical investigations and theoretical assessments. In the literature, only a few studies in which indium phthalocyanines are used as a sensitizer in SDT and aPDT applications have been reported [11,12,13,14,15]. It is important to push forward these studies, especially in the face of growing multidrug resistance.

Antimicrobial resistance is a rising global health threat, as more and more antibiotics are becoming ineffective against many bacterial and fungal infections. World Health Organization analyses show that the clinical portfolio of so-called “last choice” antimicrobial agents remains limited [31]. Moreover, few late-stage candidates show efficacy against resistant pathogens. At the same time, preclinical stages present a tendency to search for new-class agents and non-traditional approaches such as phage therapy, antimicrobial peptides, and aPDT [32,33,34,35]. Photochemical and sonochemical properties of water-soluble indium(III) Pc have not been reported in the literature; this is the first study of water-soluble sulfonated indium(III) phthalocyanine as a sensitizer in sonochemical and photochemical applications.

## 2. Experimental

### 2.1. Materials

**Figure 1:**
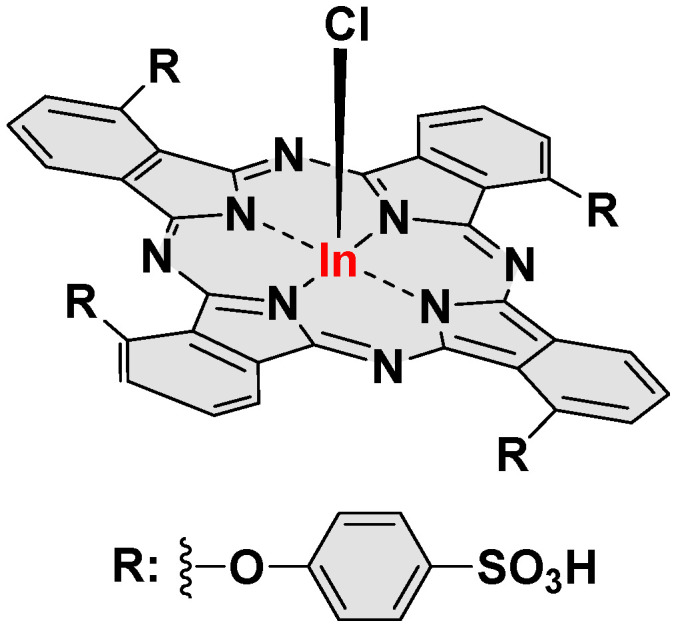
Molecular structure of the studied chloroindium(III) phthalocyanine (InPc).

1,8(11),15(18),22(25)-tetrakis(4-sulfonylphenoxy)phthalocyaninatoindium(III)-chloride (InPc) (Figure 1) and its ligand compound were prepared according to the reported procedure [36].

### 2.2. Photochemistry Measurements

#### 2.2.1. Photodegradation Quantum Yields

The photostability of the Pc was determined via the spectrophotometric method, based on changes in the compound’s spectra during irradiation. Before measurement, the Pc was dissolved in N,N-dimethylformamide (DMF, ThermoFisher, Waltham, MA, USA) and then kept at ambient temperature in the dark. Next, aliquots of the prepared solution were placed in quartz cuvettes (l = 10 mm) containing magnetic stirrers, and the cuvettes were then sealed with plugs. The samples were placed in front of a 150 W xenon lamp (Optel, Opole, Poland) equipped with an HCC-16 cutting filter to separate the visible light (>450 nm). The spectra were recorded in real time through optical fibers coupled with an OceanOptics Flame spectrophotometer and DT-MINI-2-GS light source. Quantum yield calculations were performed using Equation (1) presented below [37,38,39,40].

(1) $$\Phi =\frac{\left(c_{0}-c_{t}\right)VN_{A}}{I_{Abs}St},$$

where *c*_0_ and *c_t_* are the macrocycle concentrations, *V* is volume, *N_A_* is Avogadro’s number, *S* is area of irradiation, *t* is time of process, and *I_Abs_* is the volumetric rate of photon absorption, which is expressed by the following Equation (2):

(2) $$I_{Abs}=\int (1-10^{{-A}_{\lambda }})I_{\lambda }d\lambda ,$$

where *A_λ_* is absorbance at a chosen wavelength, and *I_λ_* is light intensity at a chosen wavelength.

#### 2.2.2. Singlet Oxygen Generation Quantum Yields Under Light Irradiation

Quantum yields of singlet oxygen generation were measured indirectly, utilizing a chemical quencher of singlet oxygen and a standard of known quantum yields as a comparison. For such comparison, 1,3-diphenylisobenzofuran (DPBF, SigmaAldrich, St. Louis, MO, USA) and unsubstituted zinc(II) phthalocyanine (ZnPc, SigmaAldrich, St. Louis, MO, USA) were chosen. The compounds were dissolved in DMF, and their solutions were kept in the dark and at ambient temperature. Before measurement, solutions of sensitizer and DPBF were mixed appropriately to achieve the desired absorbance in the stock, and finally gave concentrations of photosensitizer equal to 8.7 × 10^−6^ M and quencher equal to 3.7 × 10^−3^ M. The prepared mixture was then placed in quartz cuvettes (l = 10 mm) containing magnetic stirrers and sealed with plugs. Samples containing the chosen compounds were placed in front of the 150 W xenon lamp, equipped with an M250 monochromator (Optel, Opole, Poland) with light wavelength adjusted to the maximal absorption of InPc (690 nm at power of 0.5 mW/cm^2^), and the spectra during irradiation were recorded in real time using an OceanOptics Flame spectrophotometer equipped with optical fibers and a DT-MINI-2-GS light source. The calculations of singlet oxygen quantum yields were performed using the rates of DPBF decomposition (at λ = 417 nm) in the presence of InPc and ZnPc, according to Equation (3) presented below [37,38,41].

(3) $$\Phi_{\Delta }=\Phi_{\Delta }^{std}\frac{RI_{Abs}^{Std}}{R^{Std}I_{Abs}},$$

where $\Phi_{\Delta }^{std}$ is the quantum yield of singlet oxygen of ZnPc—the standard compound (0.67 in DMSO), *R* and *R^Std^* are the rates of DPBF degradation, and *I_Abs_* and $I_{abs}^{Std}$ are the values of light absorption of the investigated InPc and ZnPc as the standard, respectively.

### 2.3. Sonochemistry Measurements

#### 2.3.1. Sonostability

The stability of InPc during sonication was examined for its solution in DMF, prepared at ambient temperature and kept in the dark. The solution was aerated for 10 min and placed in quartz cuvettes (l = 10 mm). The cuvettes were sealed with plugs, and their spectra were recorded using a Shimadzu U-1900 spectrophotometer. Before sonication, the cuvettes were thoroughly shaken and placed in an ultrasonic apparatus equipped with a 1 MHz ultrasonic head, and the output was set at 3 W with a 40% duty cycle (the device and its equipment were built by the Institute of Fundamental Technological Research, Polish Academy of Sciences, Warsaw, Poland). The samples were sonicated for 1 min, then placed in a spectrophotometer to record spectra, then shaken again and placed in the ultrasonic apparatus for another minute until the total sonication time reached 10 min. The procedure was repeated for five independent samples, and the mean values of the obtained data were used for kinetic calculations [37,42].

#### 2.3.2. Ultrasound-Induced DPBF Decomposition

The ability of InPc to generate radicals which decompose DPBF was investigated in DMF. The solutions of both compounds were kept in the dark and at ambient temperature, mixed appropriately, then moved to quartz cuvettes (l = 10 mm) and sealed with plugs. Spectra of the samples were recorded using a Shimadzu U-1900 spectrophotometer, then the cuvettes were thoroughly shaken and placed in an ultrasonic apparatus equipped with a 1 MHz ultrasonic head with the output set at 3 W, 40% duty cycle, for 1 min. After this, the spectra were recorded, the cuvettes were shaken again, and the procedure was repeated until a total sonication time of 10 min was reached. The experiment was performed for five independent samples, and the mean values of the obtained data were used for further kinetic calculations.

### 2.4. Antimicrobial Activity

#### 2.4.1. Cell Culture

An adapted protocol described previously [43,44] was employed as follows. The experimental model included the Gram-positive bacterium methicillin-resistant *Staphylococcus aureus* (MRSA), the Gram-negative extended-spectrum *β-lactamase-producing Escherichia coli* (ESBL+ *E. coli*), and the fungus *Candida albicans* resistant to fluconazole. Bacterial strains were cultivated in Brain Heart Infusion (BHI) broth (bioMérieux, Craponne, France) at 36 ± 1 °C for approximately 24 h. *C. albicans* cultures were maintained in Sabouraud dextrose broth (Oxoid, Hampshire, UK) and incubated at 35 ± 1 °C for 24 h. All cultivation procedures were conducted under aerobic conditions. After incubation, the microbes were collected by centrifugation for a period of 15 min at 3000 rpm. The microbes were then resuspended in sterile physiological saline and diluted to a final amount of approximately 10^7^ colony-forming units (CFUs)/mL for bacteria and 10^6^ CFUs/mL for *C. albicans*.

#### 2.4.2. Photodynamic Antimicrobial Efficacy

Before irradiation, microplates containing the prepared suspensions were incubated with sensitizer (10 and 100 μM) for 30 min in conditions devoid of light. Subsequently, samples were irradiated with 660 nm red light from an LED source (manufactured by Led-Byt, Bytom, Poland) at an irradiance of 14 mW/cm^2^ to a total fluence of 50 or 100 J/cm^2^. Light power was measured at the sample plane using a calibrated optical power meter (an RD 0.2/2 radiometer manufactured by Optel, Opole, Poland). Control experiments were carried out in parallel under identical conditions but without the addition of the PS, as well as in dark conditions and in the presence of PS (dark control). After light exposure, samples were serially diluted and spread onto tryptic soy agar plates. The plates were incubated at 36 ± 1 °C for 24 h, after which the microbial kill rate was calculated by counting the number of colonies formed. As a reference, the dark control activity was chosen.

### 2.5. Computational Details

The Density Functional Theory (DFT) investigation of the InPc compound was performed using the Gaussian 16 package [39]. We studied four possible isomers generated by different orientations of the sulfonylphenoxy groups (see discussion below), and the structures of these isomers were optimized without symmetry constraints. Furthermore, for the optimized structures, vibrational frequencies were calculated in order to ensure that the optimized structures were true minima without any imaginary frequencies. Singlet structures were studied for all isomers.

In this study we used the hybrid functional B3LYP [40] along with the following combination of basis sets: Los Alamos double zeta with the corresponding pseudopotential for In, Lanl2dz [41,42], and the full-electron split-valence polarized 6-31G* basis set for all other elements, with one set of polarization functions for heavy atoms [43,44,45], the approach further referred to as B3LYP/[In:Lanl2dz;C,H,N,O,S,Cl:6-31G*]. All calculations were performed in the gas phase and with the implicit effects from H_2_O and DMF taken into account (dielectric constants 78.3553 and 37.219, respectively) using the self-consistent reaction field IEF-PCM method [46], as implemented in Gaussian 16. The UFF default model used in Gaussian 16 package with the electrostatic scaling factor α set to 1.0, was employed. The charge analysis was performed using the Natural Bond Orbital (NBO) scheme as implemented in Gaussian 16 [47]. Molecular orbitals (MOs) for the optimized structures were computed using the implicit H_2_O effects at the B3LYP/[In:Lanl2dz;C,H,N,O,S,Cl:6-31G*] level.

Furthermore, the HOMO (highest occupied molecular orbital) and LUMO (lowest unoccupied molecular orbital) energies were used to calculate the global reactivity parameters [48,49,50,51,52,53] of the compounds under investigation (see Equations (4)–(11) below). Equations (4) and (5) were used for the ionization potential (*IP*) and electron affinity (*EA*) values:

*IP* = −*E*_HOMO_; (4)

*EA* = −*E*_LUMO_. (5)

Global hardness *η* and global electronegativity X were computed by Equations (6) and (7), respectively:

*η* = [*IP* − *EA*]/2 = −[*E*_LUMO_ − *E*_HOMO_]/2; (6)

X = [*IP* + *EA*]/2 = −[*E*_LUMO_ + *E*_HOMO_]/2. (7)

Global electrophilicity *ω* was calculated by using Equation (8):

*ω* = *μ*^2^/2*η*, (8)

where *μ* = [*E*_HOMO_ + *E*_LUMO_]/2 is the chemical potential of the system, and *η* is the global hardness. 

The global softness *σ* value was calculated using Equation (9) (*η* is the global hardness):

*σ* = 1/2*η*. (9)

*ω*^−^, the propensity to donate electrons, was computed using Equation (10) (*η* is the global hardness):

*ω*^−^ = (*E*_HOMO_)^2^/(2*η*). (10)

*ω*^+^, the propensity to accept electrons, was computed using Equation (11) (*η* is the global hardness):

*ω*^+^ = (*E*_LUMO_)^2^/(2*η*). (11)

Molecular structures and frontier MOs were visualized using Avogadro visualization software, version 1.1.1 [54,55]. Gabedit, a graphical user interface for computational chemistry software, version 2.5.1, was used to calculate the GRP values [56]. Multiwfn software, version 3.8 [57], was used for the molecular electrostatic potential (MEP) [58,59] analysis. VMD for WIN64, version 1.9.4a53, was used to visualize the MEP plot [60].

## 3. Results and Discussion

### 3.1. Photochemical and Sonochemical Properties

**Figure 2:**
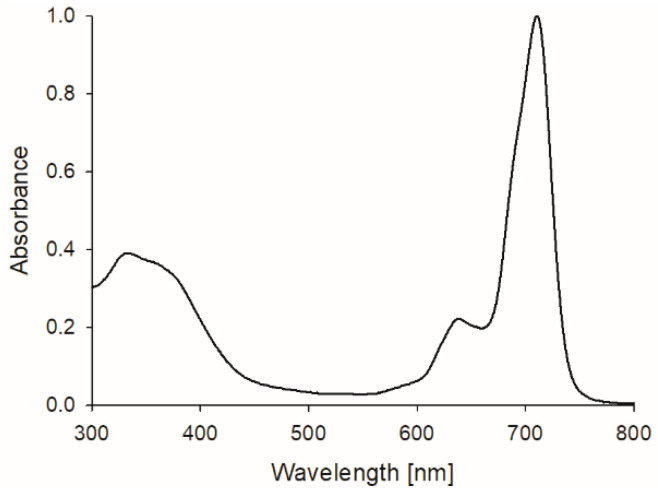
UV–Vis absorption spectrum of the InPc in DMF.

The studied InPc reveals absorption bands typical for phthalocyanine moieties, with the Soret band between 300 and 450 nm and the Q-band between 600 and 750 nm (Figure 2), which are associated with π–π* electron transitions between respective occupied molecular orbitals and the lowest unoccupied (Soret band) or degenerated and non-degenerated (Q-band) molecular orbitals [45]. Although the synthesized phthalocyanine complex exhibits reasonably good solubility in aqueous media owing to its hydrophilic substituents, it displays a strong tendency to form photoinactive H-type aggregates in pure water due to intermolecular π–π stacking and hydrophobic interactions. Since aggregation severely quenches the excited states and diminishes both fluorescence and singlet oxygen generation efficiencies, it was essential to conduct the photophysical and photochemical measurements in an organic solvent. Consequently, all related optical studies were performed in DMF to ensure the macrocycle remained in its fully monomeric state, thereby allowing an accurate evaluation of its intrinsic photoactive properties. A high molar extinction coefficient of log_10_ε = 4.56 of the InPc was calculated at the maximum of the Q-band (DMF). These transitions result in absorption maxima located in the red-light region, which is considered the most suitable for PDT. Red light combines the deepest tissue penetration within the visible spectrum with sufficient photon energy to trigger the photodynamic effect [3,46].

**Figure 3:**
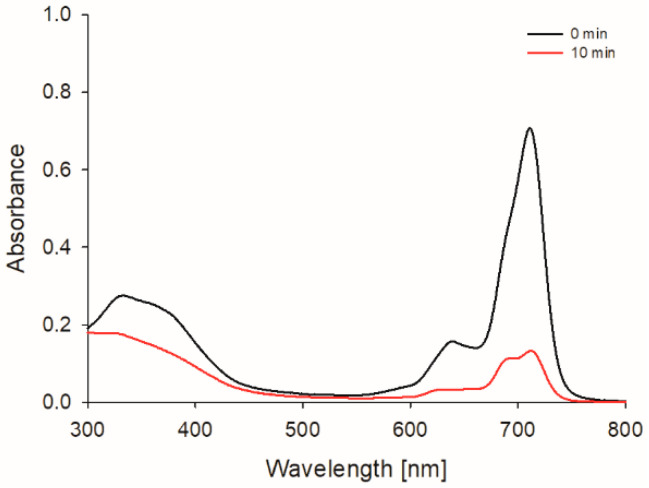
Changes in the absorption spectrum of the InPc in DMF after irradiation with visible light.

As shown in Figure 3, the exposition of InPc to the visible light (>450 nm), while dissolved in DMF, reveals a high rate of InPc decomposition. During the process, a phototransformation was observed, manifested by Q-band splitting and a change in the spectral band curvature. Following irradiation, the disappearance of the Q-band was noticed. The splitting of the Q-band is not usual for phthalocyanine. Numerous reports on photobleaching with a vanishing unchanged (in shape) Q-band as characteristic for phthalocyanines have been published [46,47,48,49]. Q-band splitting in the InPc studied here might be as a result of lowering the symmetry of the macrocycle by opening one of the isoindole units of the ring during the photodecomposition process, with simultaneous core preservation. The formed open-isoindole macrocycle seems to be relatively stable.

The calculated quantum yield of photodecomposition at the level of 10^−4^ (Table 1) implies moderate–high stability of the macrocycle according to the classification published by Dilber et al. [50]. The Φ_P_ value calculated here is one magnitude lower than reported before for a sample dissolved in DMSO (2.0 × 10^−5^), and one magnitude higher than for the water solution (1.9 × 10^−3^) [36]. Pcs can interact with solvent molecules, finally leading to an increase or decrease in their stability. As is well known, DMSO coordinates to the central metal ion, thereby inhibiting the stacking of Pc molecules into aggregates [51,52]. Słota and Dyrda have studied the photostability of different metal complexes of phthalocyanine and concluded that the most important factor is the structure of the complex. The most unstable complexes are those bearing large metal ions, like indium(III), in the coordination center. The ion moves above the ring, which causes the deformation of the plane phthalocyanine macrocyclic ring. Additional torsions lower the photostability of the molecule. Moreover, these authors reported that DMSO provides a much stronger stabilizing effect than DMF [53], which is in agreement with the data presented here. Interestingly, the values of photodecomposition quantum yields for InPc are comparable to those of unsubstituted chloroindium(III) phthalocyanine reported before [54]. This enables us to conclude that the most important factor varying the photostability for our sensitizer is the coordinated chloroindium(III) ion, while the substituents seem to possess a negligible impact.

**Figure 4:**
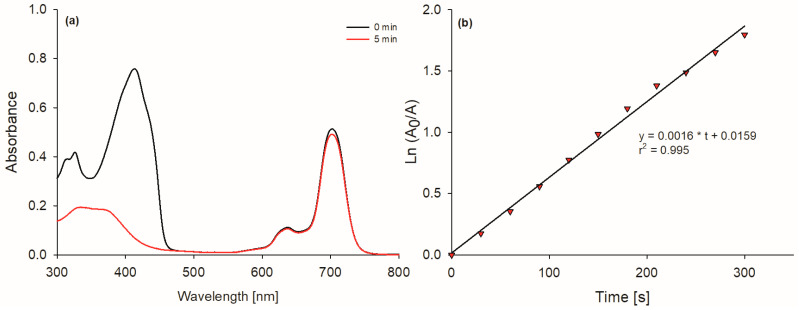
(**a**) Spectra of InPc mixture with DPBF before and after irradiation; (**b**) kinetic curve of the process.

Surprisingly, we noticed moderate–high photostability with a Φ_P_ value at the level of 10^−4^ (Table 1, Figure 4). It is unusual that an efficient singlet oxygen generator presents high stability [56]. The quantum yield of singlet oxygen formation (Φ_Δ_) reached up to 0.82 ± 0.04, compared with ZnPc. Interestingly, the photostability of InPc was about 1.5-fold lower than that of the reference ZnPc, corresponding approximately to a 1.5-fold higher singlet oxygen quantum yield. The kinetics of the process correspond to the first-order equation, as shown in Figure 4b. The closed-shell chloroindium(III) cation is a major determinant leading to high singlet oxygen formation ability. It is linked with the spin–orbit coupling phenomenon of large metal ions and their impact on the photochemistry of porphyrinoids [13,56,57]. On the other hand, the introduction of non-peripheral 4-sulfophenyl substituents with an electron-withdrawing character might increase the value of Φ_Δ_. Such an effect and its impact on the value of Φ_Δ_ has been reported before [58,59].

**Figure 5:**
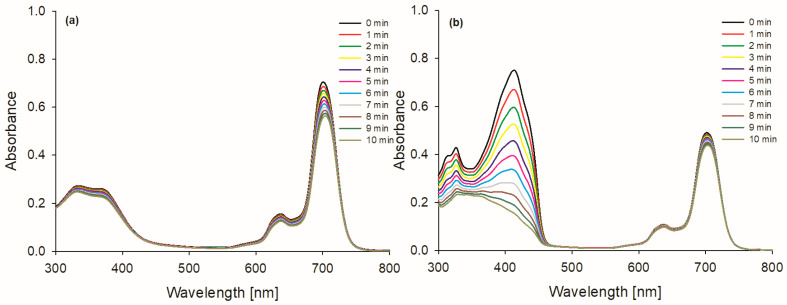
(**a**) Spectra changes in InPc during sonication; (**b**) spectra changes in InPc with DPBF during sonication.

The stability of InPc under exposition to ultrasounds at parameters of 3 W and a 40% duty cycle was evaluated. The results obtained indicate that the decomposition process was of the first order from a kinetic point of view. Study of the recorded absorption spectra (Figure 5a) during the sonodecomposition process might suggest that under ultrasounds, the compound decomposed to lower-weight molecules (no absorption from the dye), similar to photobleaching, which might be termed sonobleaching.

The calculated half-life time for InPc (Table 2) of 31 min in comparison to sonosensitizers reported before enables us to conclude that the studied molecules reveal moderate sonostability [37,42,46,60,61,62]. Interestingly, Wysocki et al. have reported SiPcs with well-developed axial substituents and simultaneously found that molecules not possessing peripheral substituents showed a higher potential to decompose DPBF under ultrasound exposition. At the same time, it was shown to have an extremely high sonostability, with a half-life time of 7737 min [42]. Taking into account the reasonable impact of coordinated metal ions, it can be assumed that axial substituents also possess reasonable stabilization ability.

**Figure 6:**
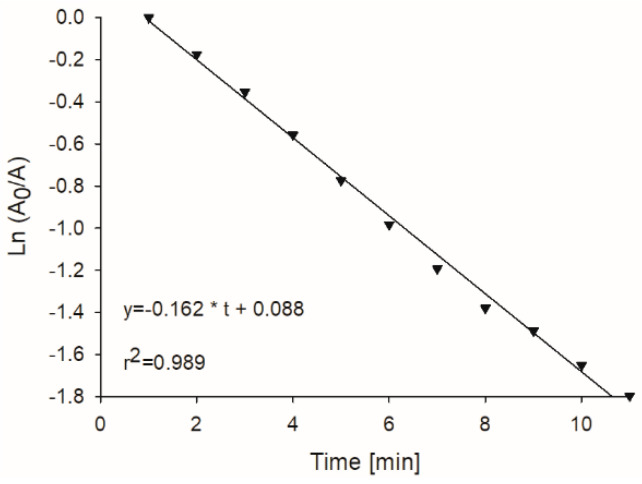
The kinetic curve for ultrasound-induced DPBF decomposition by InPc.

The evaluated sonosensitizer InPc showed the best activity in DPBF-mediated decomposition by excitation of the sensitizer with ultrasounds (t_0.5DPBF_ = 3.7 ± 0.3 min, Figure 5b) within previously reported sonosensitizers by our group [37,42,46,62]. The DPBF decomposition process appears to follow first-order kinetics (Figure 6). The significant impact of apical substituents on the sonosensitizer sonostability can be verified by the comparison of the InPc studied here with SiPc reported previously [42]. The structure of InPc enables unrestricted access of ROS to the macrocyclic ring in contrast to SiPc, with more developed apical substituents [42]. Moreover, InPc forms ROS more efficiently than SiPc [42].

### 3.2. Photobiological Evaluation

The potential of InPc in the photodynamic inactivation of microbes revealing resistance to conventional antibiotic treatment, was studied. First, the dark activity of the studied compound was assessed, revealing no significant reduction in the viability of the tested microbes (log_10_ reduction in bacterial growth below 0.5). The evaluated PS showed a high ability to combat MRSA and the fungus *C. albicans* resistant to fluconazole, whereas the extended-spectrum *β-lactamase-producing E. coli* was generally unaffected by the proposed photodynamic procedure. Only with a higher dosimetry (PS concentration 100 μM and fluence rate of 100 J/cm^2^) was a slight growth inhibition noticed (Table 3).

Considering full dosimetry parameters, the observed growth inhibition rate for InPc against MRSA (>4.04 log_10_; 50 J/cm^2^) was stronger than the one reported before by Wysocki et al. for axially substituted SiPc (>5.25 log_10_; 150 J/cm^2^) [42] or zinc(II) phthalocyanines (3.47 log_10_ and 0.92; 150 J/cm^2^) [42,46]. An interesting phenomenon has been reported by Sifeko et al. that the larger number of positive charges (quaternized 4-pirydoxyl substituents) present in indium(III) phthalocyanines significantly increases their photodynamic antimicrobial potential. Moreover, authors have observed that the position of substitution (peripheral vs. non-peripheral) does not significantly affect the photodynamic activity of indium(III) phthalocyanines [63]. The same group also reported a high microbial photodynamic inactivation potential of indium(III) phthalocyanine due to it bearing more developed substituents with positive charges [64]. These studies indicate that regarding the photoactivity of indium(III) Pc complexes, the most important factor is the number of positive charges. On the other hand, Domínguez et al. have reported high photodynamic activity of axially substituted ruthenium phthalocyanine complexes against MRSA (4.90 log_10_; 30 J/cm^2^) [65]. It might be assumed that the axial substituent varies in antimicrobial activity against MRSA. Not without meaningful impact are sulfonic groups attached at the periphery of the studied InPc; Sarabando et al. reported high anti-MRSA activity of sulfonated porphyrins [66]. Different behavior in the photodynamic inactivation of *C. albicans* resistant to fluconazole was observed. Only in the higher concentration was the photodynamic killing effect observed, with a log_10_ reduction in fungi growth of 4.08 (Table 3). Interestingly, it is well known that cationic macrocycles tightly bind to fungal cells in contrast to anionic ones, where activity is usually detected for low concentrations of cationic PS [67,68]. This might be an explanation for the absence of activity observed here for low concentrations of InPc. Particularly important when predicting the potential activity of a PS is the analysis and characterization of the cell wall and membrane of the pathogen against which it is intended to act. Numerous publications indicate that Gram-positive bacteria are more sensitive than Gram-negative bacteria to most investigated PSs [69,70]. However, there are numerous exceptions to this rule. Unlike *E. coli*, MRSA is characterized by the presence of a single bacterial membrane. This membrane provides considerably weaker protection against PS penetration. At the same time, when considering defense mechanisms, differences in the structure and substrate specificity of individual efflux pumps characteristic of each studied strain should be taken into account [71]. The primary efflux pump relevant from the PDT perspective for *S. aureus* is the NorA pump. This pump is highly selective for cationic macrocycles [72]. In the InPc structure studied here, free -SO_3_H groups can readily transform into -SO_3_^−^. This form can be practically ignored by the indicated transporter. In the case of *E. coli*, there is a powerful AcrAB-TolC complex. It is capable of transporting much larger molecules than the NorA transporter [73]. Although the incorporation of the PS into the cell is not critical for its antimicrobial activity, the lack of a positive charge, allowing the compound to bind electrostatically to the membrane, may play a particularly important role. This, consequently, leads to reduced activity against *E. coli*.

### 3.3. DFT Studies

**Figure 7:**
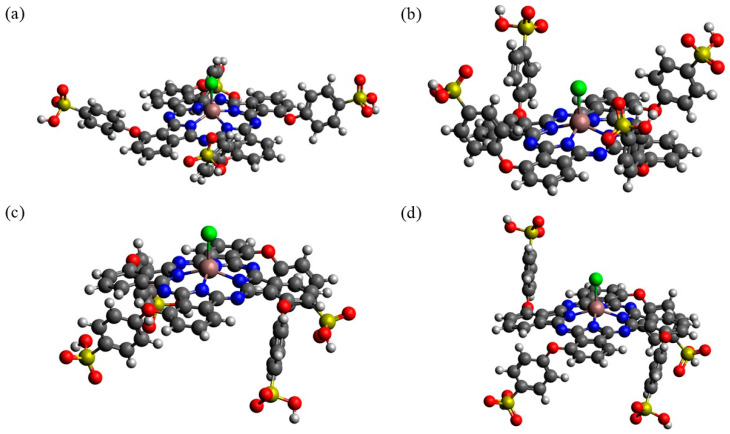
Isomers 1 (**a**), 2 (**b**), 3 (**c**), and 4 (**d**) of the InPc optimized at the B3LYP/[In:Lanl2dz;C,H,N,O,S,Cl:6-31G*] level in the implicit water. Color coding: dark gray for C, light gray for H, dark blue for N, yellow for S, red for O, brownish for In.

As mentioned in the Section 2.5, we studied four different isomers of the InPc compound whose starting structures were obtained by various orientations of the substituents of the phthalocyanine moiety. The results of optimizations with implicit effects from water are shown in Figure 7, and important energetic characteristics of the four isomers are given in Table 4 (below, we focus predominantly on the results obtained in the implicit water; for the TDDFT results, both implicit water and DMF results are provided). Analysis of the results provided in Table 4 and in Figure 7 shows the following features. (i) Interestingly, in the lowest-lying isomer 4, one substituent group is oriented up, and three substituent groups are oriented down (Figure 7d), whereas in the closest in energy isomer 2, all four substituents are oriented up (Figure 7b).

(i) In the highest-lying isomer 1 (Figure 7a), all four substituent groups are in some way oriented up but essentially are close to the Pc moiety plane, and in isomer 3 (Figure 7c), all four substituent groups are oriented down, although only two of them are directed down more vertically, similar to isomer 4. Also, all four isomers show noticeable out-of-plane coordination of the InCl group to the nitrogens of the Pc moiety (see below, more detailed discussion for isomers 2 and 4). (ii) Isomers 4 and 2 are very close in energy to each other, energy differences being −0.18–0.16 kcal/mol, with isomer 2 being lower in energy in the implicit water by 0.18 kcal/mol. Essentially, these two isomers could be considered as degenerate. Isomers 3 and 1 are higher in energy compared to isomer 4, by 0.82–3.35 kcal/mol, although these differences are not very significant. This implies that these four isomers might coexist in water or DMF solutions; thus, easy rotation of the sulfonylphenoxy groups causes facile transfers between different InPc isomers and easy accommodation of InPc molecules to the environment. (iii) Also, as can be seen in Table 4, the HOMO/LUMO and TDDFT gaps of these four isomers are close to each other. Isomers 4 and 2 have the HOMO/LUMO gaps of 2.08 and 2.08–2.09 eV, respectively, whereas isomers 3 and 1 have the HOMO/LUMO gaps of 2.01–2.03 and 2.08–2.09 eV, respectively. Furthermore, the TDDFT gaps of these isomers (calculated with ωB97XD functional) are also close to each other; thus, for isomer 4 these gaps are 1.80–1.87 eV, and for isomer 2 they are 1.80–1.88 eV, whereas for isomer 3 they are 1.79–1.88 eV and for isomer 1 they are 1.76–1.85 eV. This implies very similar optical properties of these isomers (see discussion below). It is interesting to notice that while the HOMO/LUMO gaps are the same or almost the same in the gas phase and in the implicit solvents, the TDDFT gaps slightly decrease from the gas phase to the implicit solvents, by 0.07–0.09 eV. (iv) The dipole moments of different isomers differ significantly due to different orientations of the sulfonylphenoxy groups. Thus, for isomer 4, the dipole moments have values of 4.04–5.34 D, somewhat increasing from the gas phase to the implicit solvents, especially water, whereas for isomer 2, they are noticeably larger, being 8.76 D in the gas phase and 16.27 and 16.62 D in the implicit water and DMF, respectively. Furthermore, for isomer 3, the dipole moments have moderate values of 3.28–4.81 D, steadily increasing from the gas phase to the implicit DMF. Finally, for isomer 1, they again have high values of 11.68–16.31 D, increasing from the gas phase to the implicit water and then slightly dropping to the implicit DMF. These significant dipole moment values imply the possibility of noticeable interactions of the InPc molecules with polar molecules of solvent media, intracellular media, and with various oxygen-containing species; therefore, they might affect the performance of InPc in the photo- and sonodynamic therapy processes.

**Figure 8:**
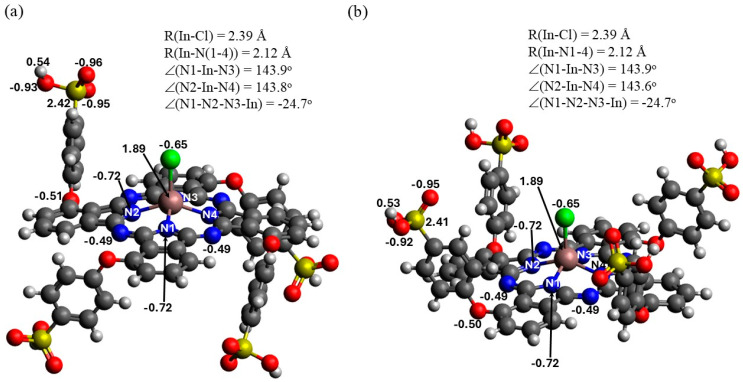
Isomers 4 (**a**) and 2 (**b**) of InPc optimized at the B3LYP/[In:Lanl2dz;C,H,N,O,S,Cl:6-31G*] level in the implicit water. Selected bond distances shown in angstroms, selected bond angles and dihedral angles shown in degrees. NBO charges on selected atoms shown in bold. Color coding: dark gray for C, light gray for H, dark blue for N, yellow for S, red for O, brownish for In.

Figure 8 compares the geometries of the lowest-lying isomer 4 and the next by energy, isomer 2, optimized in implicit water. It can be seen that despite the different orientations of the sulfonylphenoxy group geometries of the Pc core in both, the cases are essentially the same. The In-Cl unit protrudes significantly from the Pc cavity, as can be noticed from the values of valence angles, ∠(N1-In-N3) and ∠(N2-In-N4), by ca. 36° smaller than 180°, and dihedral angles, ∠(N1-N2-N3-In), with the absolute value ca. 25°. This would make the In-Cl unit more accessible for interactions with various species in the solution media.

Analysis of NBO charges on selected atoms shown in Figure 8 for the isomers 4 and 2 shows the following features. (i) The charges in the Pc moiety are the same for both isomers. Thus, the In centers have significant positive charges of 1.89e, whereas the Cl centers carry significant negative charges of −0.65e. The pyrroles’ nitrogen have noticeable negative charges, around −0.72e, and the meta-nitrogen of the Pc core carry noticeable negative charges as well, around −0.49e. (ii) The charges in the sulfonylphenoxy groups are also very close in both isomers; thus, the oxygens connecting these groups with the Pc core have significant negative charges, between −0.50 and −0.51e, the sulfurs of the sulfonylphenoxy groups have high positive charges, between 2.41 and 2.42e, and the oxygens of the sulfonyl groups have significant negative charges, between −0.95 and −0.96e for the =O-type oxygens and between −0.92 and −0.93e for the -OH-type oxygens. Also, hydrogens of the OH-groups carry significant positive charges, around 0.53–0.54e.

These charges imply that the InPc should strongly interact with polar solvent molecules through both dipole–dipole and hydrogen bonding, thus being easily soluble in water and other polar solvents. Moreover, they imply that this compound should interact strongly with polar parts of proteins and other biologically important molecules, as well as with various O-containing species, through both dipole–dipole and hydrogen bonding.

**Figure 9:**
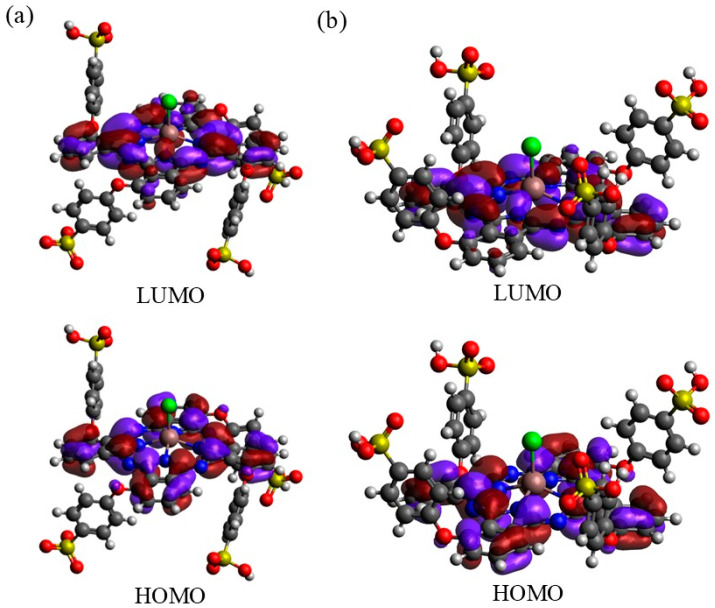
Frontier MOs of the isomers 4 (**a**) and 2 (**b**) of InPc computed at the B3LYP/[In:Lanl2dz;C,H,N,O,S,Cl:6-31G*] level in the implicit water.

Analysis of the frontier MOs of the isomers 4 and 2 presented in Figure 9 shows that in both cases, only the Pc core contributes to both HOMO and LUMO, without any contributions from the sulfonylphenoxy groups or the In-Cl units. This is in agreement with the experimental spectral data showing that the InPc investigated demonstrates typical absorption bands of phthalocyanine moieties (see also the discussion of TDDFT results below).

**Figure 10:**
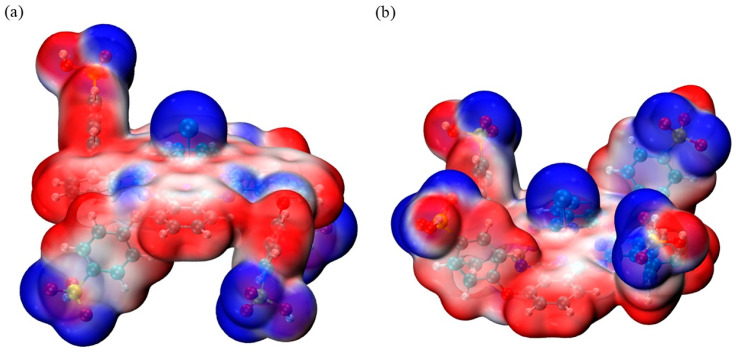
Plots of MEP for the isomers 4 (**a**) and 2 (**b**) of InPc computed at the B3LYP/[In:Lanl2dz;C,H,N,O,S,Cl:6-31G*] level in implicit water.

The molecular electrostatic potential plots provided in Figure 10 show the following features. (i) For both isomers, there are noticeable accumulations of positive MEP (shown by red color) over the Pc cores and hydrogens of the phenyl moieties of the sulfonylphenoxy groups, as well as hydrogens of the OH-groups of the sulfonylphenoxy moieties. (ii) There are significant accumulations of negative MEP (shown by blue color) on the Cl-centers and on the oxygens of the sulfonyl groups, as well as some accumulation of negative MEP over the phenyl units of the sulfonylphenoxy groups. Moreover, an essentially neutral potential can be seen over the Pc aromatic rings. These results imply that the InPc should strongly interact with polar solvent molecules through both dipole–dipole and hydrogen bonding, thus being easily soluble in water and other polar solvents. Also, it should strongly interact with polar parts of proteins and other biologically important molecules, as well as with various O-containing species, through both dipole–dipole and hydrogen bonding.

The computed values of the global reactivity parameters, eV, namely ionization potential (*IP*), electron affinity (*EA*), global electronegativity (*X*), global hardness (*η*), chemical potential (*μ*), global softness (*σ*), global electrophilicity (*ω*), propensity to donate electrons (*ω*^−^), and propensity to accept electrons (*ω^+^*), computed using the FMO energies (Table 4) according to Equations (4)–(11) (see Section 2.5), are presented in Table 5.

Analysis of the GRP values for both isomers 4 and 2, provided in Table 5, shows the following features: (i) Both isomers have significant IP and EA values, around 5.49 and 3.41 eV, respectively. This suggests that the InPc compound should be considered as a relatively poor electron donor and a good electron acceptor. (ii) Both isomers have noticeable values of the HOMO/LUMO gap, 2.08 eV, which, along with significant absolute values of chemical potential, ca. −4.45 eV, suggests that InPc should be considered to be kinetically stable. (iii) Furthermore, the global hardness values for both isomers are not very high, ca. 2.08 eV, and the global softness values are ca. 0.48 eV^−1^, which could be considered as a moderately high value. This suggests that the InPc compound should be considered as moderately reactive. (iv) Next, the global electronegativity and global electrophilicity values for both isomers are significant, ca. 4.45 and 4.76 eV, respectively, which, along with the significant IP and EA values, implies that InPc should be considered as a reasonably good electron acceptor. (v) Finally, relatively high values of the propensity to accept electrons, ca. 2.80 eV, further support the suggestion that InPc should be a good electron acceptor. However, significantly higher values of the propensity to donate electrons, ca. 7.24–7.25 eV, would suggest that this compound should be considered as a good electron donor as well, when a proper electron acceptor, such as oxygen-containing reactive species, is present. This seeming contradiction between the high IP values and high electron donation propensity values can be explained by structural electron delocalization and metal coordination, which take place in the metalated Pc system, where electron donation occurs without directly removing high-energy electrons that account for the ionization potential [74,75].

Therefore, from the GRP analysis, it can be seen that InPc would easily interact in the solution phase with electron-excess species such as various free radicals. Also, it should be able to interact with electrophilic species such as hydroxyls and other O-containing free radicals. This suggests that this compound might be considered to be a good free radical inhibitor.

Analysis of the TDDFT results for the excited state 1 of the isomer 4 provided in Table 6 shows the following features. (i) The excited state is mostly contributed by the HOMO→LUMO transition, both in the implicit water and DMF, with % contributions of 93.16 and 94.62, respectively. The oscillator strengths of this excited state are significant in both solvents, ca. 0.60 and 0.63, respectively. (ii) Next, the wavelength obtained in the implicit DMF, 690.34 nm, is very close to the experimental value (see Figure 1) of 712 nm. These results further confirm the experimental data, showing the InPc to be a good photochemical agent.

## 4. Conclusions

In summary, the photodynamic antimicrobial activity of indium(III) phthalocyanine bearing *p*-sulfonylphenoxy groups at the non-peripheral positions (InPc) was evaluated. The evaluated InPc exhibited high rates of singlet oxygen generation (Φ_Δ_ = 0.82 ± 0.04). Furthermore, exposure to ultrasound resulted in substantial DPBF decomposition. Simultaneously, the studied macrocycle showed moderate–high stability when exposed to either light or ultrasound. The DFT results imply that four isomers of the InPc might coexist in water or DMF solutions and thus, easy rotation of the sulfonylphenoxy groups causes facile transfers between different InPc isomers and easy accommodation of InPc molecules to the environment. Analysis of the frontier MOs of the InPc isomers 4 and 2 shows that in both cases, only the Pc core contributes to both HOMO and LUMO, without any contributions from the sulfonylphenoxy groups or the In-Cl units. This is in agreement with the experimental spectral data showing that the InPc studied demonstrates typical absorption bands of phthalocyanine moieties. The MEP analysis results imply that the InPc should strongly interact with polar solvent molecules through both dipole–dipole and hydrogen bonding, thus being easily soluble in water and other polar solvents. Also, it should noticeably strongly interact with polar parts of proteins and other biologically important molecules, as well as with various O-containing species, through both dipole–dipole and hydrogen bonding. The GRP analysis results imply that InPc would easily interact in the solution phase with electron-excess species such as various free radicals, and it should be able to interact with electrophilic species such as hydroxyls and other O-containing free radicals. The TDDFT results further confirm the experimental data, showing InPc to be a good photochemical agent. The potential of InPc in antimicrobial photodynamic studies is evident, especially with regard to its high potential in combating MRSA and the fungus *C. albicans* resistant to fluconazole, which was observed with log_10_ reductions in microbial growth of >4.04 (10 μM and 50 J/cm^2^) and 4.08 ± 0.29 (100 μM and 50 J/cm^2^), respectively.

## Figures and Tables

**Table 1:** Values of Φ_Δ_ and Φ_P_ for InPc and standard ZnPc.

Compound	Solvent	10^6^ Φ_P_	Φ_Δ_
InPc	DMF	527.6 ± 13.1	0.82 ± 0.04
ZnPc	DMF	10.2 [48]	0.56 [55]

**Table 2:** Kinetic parameters of InPc and DPBF decomposition upon sonication [1 MHz] (parameters of sensitizer decomposition under ROS formation in the experiment with DPBF are given in brackets).

Compounds	k (min^−1^)	t_0.5_ (min)	Ln (A)
DPBF	0.024 ± 0.001	28.3 ± 0.3	−0.016 ± 0.038
InPc	0.020 ± 0.002	31.6 ± 2.1	0.002 ± 0.003
DPBF with InPc	0.188 ± 0.012 [0.011 ± 0.012]	3.7 ± 0.3 [60.8 ± 0.3]	−0.088 ± 0.012 [−0.001 ± 0.012]

**Table 3:** Log_10_ values of the reduction in microbes under irradiation with red light in the presence of the evaluated photosensitizer.

	50 J/cm^2^		100 J/cm^2^	
	10^−4^ M	10^−5^ M	10^−4^ M	10^−5^ M
*MRSA*	>4.04	>4.04	>4.04	>4.04
*E. coli (ESBL+)*	0.27 ± 0.25	0.27 ± 0.19	2.64 ± 0.36	0.49 ± 0.28
*C. albicans*	3.91 ± 0.32	−0.13 ± 0.19	4.08 ± 0.29	0.58 ± 0.17

**Table 4:** Selected energetic parameters for four isomeric structures obtained for the InPc compound with the B3LYP/[In:Lanl2dz;C,H,N,O,S,Cl:6-31G*] approach in the gas state//water//DMF.

Isomer	E_0_, A.U.	E_0_+ZPE, A.U.	E(HOMO/ LUMO), A.U.	ΔE_H/L_, eV	ΔE_TD-B3LYP_, eV	ΔE_TD-ωB97XD_, eV	μ, D	ΔE, kcal/ mol
1	−5849.492 9i ^a^// −5849.555 4i// −5849.553 3i	−5848.683// −5848.747 −5848.745	−0.200/−0.125// −0.196/−0.122// −0.196/−0.122	2.03// 2.01// 2.01	1.91// 1.84// 1.83	1.85// 1.77// 1.76	11.68// 16.31// 16.04	3.35// 1.08// 1.53
2	−5849.497// −5849.557 8i, 4i// −5849.555	−5848.687// −5848.749// −5848.747	−0.208/−0.131// −0.202/−0.125// −0.202/−0.125	2.09// 2.08// 2.08	1.97 1.91// 1.90	1.88// 1.81// 1.80	8.76// 16.27// 16.62	0.15// −0.18// 0.16
3	−5849.495 52i// −5849.555 10i// −5849.554	−5848.686// −5848.748// −5848.746	−0.210/−0.134// −0.201/−0.125// −0.202/−0.125	2.09// 2.08// 2.08	1.96// 1.91// 1.90	1.88// 1.81// 1.79	3.28// 4.64// 4.81	1.24// 0.82// 1.13
4	−5849.497// −5849.556// −5849.556 6i, 2i	−5848.687// −5848.748// −5848.747	−0.208/−0.132// −0.202/−0.125// −0.202/−0.125	2.08// 2.08// 2.08	1.96 1.91// 1.90	1.87// 1.81// 1.80	4.04// 5.34// 4.92	0.0// 0.0// 0.0

^a^ These numbers are imaginary frequencies which are generally small (except for isomer 3 in the gas phase) and cannot be removed by further optimization. Attempts to remove them usually do not decrease the energy significantly; therefore, the structures are presented with these small imaginary frequencies.

**Table 5:** The calculated GRPs for the InPc isomer 4 and 2 (eV; eV^−1^ for global softness), computed at the B3LYP/[In:Lanl2dz;C,H,N,O,S,Cl:6-31G*] level in the implicit water.

Isomer	*IP*	*EA*	*Gap*	*X*	*η*	*μ*	*σ*	*ω*	*ω* ^+^	*ω* ^−^
**4**	5.485	3.408	2.08	4.447	2.077	−4.447	0.481	4.760	2.796	7.243
**2**	5.486	3.411	2.08	4.448	2.075	−4.448	0.482	4.768	2.802	7.251

**Table 6:** TDDFT results (Q-band) for the ground state InPc calculated at the TD-ωB97XD/[In:Lanl2dz;C,H,N,O,S,Cl:6-31G*] level with the implicit water/DMF for comparison.

Excited State	Orbital Transitions with Contributions, %	Transition Energy, eV	λ, nm	Oscillator Strength
1	HOMO→LUMO, 93.16 HOMO-5→LUMO+1, 4.44// HOMO→LUMO, 94.62 HOMO-5→LUMO+1, 4.31	1.81// 1.80	684.91// 690.34	0.5985// 0.6295

## Data Availability

The data presented in this study are available upon request from the corresponding author.
